# Supplementary material for: Identifying antimicrobial peptides using word embedding with deep recurrent neural networks
Source: Bioinformatics. 2018 Nov 10;35(12):2009–16. doi: 10.1093/bioinformatics/bty937 (PMC6581433; doi:10.1093/bioinformatics/bty937)
Supplement: bty937_Supplementary_Data [file bty937_supplementary_data.pdf]

Supplementary Material

Table 1: Primary Bacteriocin dataset. Comparison between word2vec and trigram representation. SVM:Support Vector Machine; LogReg: logistic regression; DT: decision tree; RF: random forest, w2v + RNN: Recurrent Neural Network with word2vec representation.

	**Mean Precision**	**Mean Recall**	**Mean F_1**
**trigram+SVM**	0.875 ± 0.001	0.808 ± 0.002	0.838 ± 0.001
**trigram+LogReg**	0.864 ± 0.002	0.837 ± 0.002	0.846 ± 0.001
**trigram+DT**	0.767 ± 0.002	0.735 ± 0.002	0.747 ± 0.001
**trigram+RF**	0.838 ± 0.001	0.791 ± 0.001	0.812 ± 0.001
**w2v averaged+SVM**	0.889 ± 0.0007	0.848 ± 0.001	0.867 ± 0.0008
**w2v averaged+LogReg**	0.848 ± 0.001	0.817 ± 0.001	0.831 ± 0.0009
**w2v averaged+DT**	0.825 ± 0.001	0.813 ± 0.002	0.738 ± 0.002
**w2v averaged+DRF**	0.838 ± 0.001	0.791 ± 0.001	0.817 ± 0.001
**BLAST**	0.972 ± 0.0006	0.506 ± 0.002	0.663 ± 0.002
**HMMER**	**0.981 ± 0.0002**	0.757 ± 0.0001	0.852 ± 0.0003
**w2v + RNN**	0.898 ± 0.003	**0.883 ± 0.003**	**0.889 ± 0.001**

Table 2: Second Bacteriocin dataset.

	**Mean Precision**	**Mean Recall**	**Mean F_1**
**SVM**	0.902 ± 0.001	0.835 ± 0.001	0.865 ± 0.0009
**LogReg**	0.891 ± 0.001	0.871 ± 0.001	0.878 ± 0.001
**DT**	0.806 ± 0.002	0.767 ± 0.002	0.782 ± 0.001
**RF**	0.858 ± 0.001	0.792 ± 0.001	0.822 ± 0.001
**BLAST**	0.909 ± 0.002	0.504 ± 0.002	0.645 ± 0.001
**HMMER**	**0.985 ± 0.0001**	0.757 ± 0.0001	0.854 ± 0.0003
**w2v + RNN**	0.924 ± 0.002	**0.898 ± 0.001**	**0.909 ± 0.001**

Table 3: Third Bacteriocin dataset.

	**Mean Precision**	**Mean Recall**	**Mean F_1**
**SVM**	0.938 ± 0.001	0.898 ± 0.001	0.916 ± 0.0009
**LogReg**	0.916 ± 0.001	0.891 ± 0.001	0.902 ± 0.0007
**DT**	0.887 ± 0.001	0.856 ± 0.001	0.869 ± 0.001
**RF**	0.889 ± 0.001	0.878 ± 0.001	0.882 ± 0.001
**BLAST**	0.747 ± 0.004	0.504 ± 0.002	0.599 ± 0.002
**HMMER**	**0.992 ± 0.0001**	0.757 ± 0.0001	0.857 ± 0.0003
**w2v + RNN**	0.937 ± 0.002	**0.921 ± 0.002**	**0.928 ± 0.002**
